# General perception of the diversification of child-rearing environments in Japan

**DOI:** 10.3389/fsoc.2026.1674416

**Published:** 2026-03-06

**Authors:** Kazushi Maruya, Rokuro Tabuchi, Junji Watanabe, Chihiro Hosoda

**Affiliations:** 1 Communication Science Laboratories, NTT, Inc., Atsugi, Kanagawa, Japan; 2 Department of Sociology, Sophia University, Chiyoda-ku, Tokyo, Japan; 3 Department of Human-Social Information Sciences, Graduate School of Information Sciences, Tohoku University, Sendai, Miyagi, Japan; 4 Division of Brain Sciences, Institute of Development, Aging and Cancer, Tohoku University, Sendai, Miyagi, Japan

**Keywords:** child-rearing, diversity, family form, Japanese family form, public image

## Abstract

Understanding how child-rearing environments and family dynamics evolve in response to socioeconomic change and shifting cultural values is a central question in sociology. The Second Demographic Transition (SDT) framework explains many of these transformations in Western societies; however, its relevance to East Asia—particularly Japan—remains uncertain due to the region's distinctive family norms and institutional settings. In Japan, traditional family models continue to shape parenting and caregiving, creating unequal burdens on mothers despite recent institutional reforms. This study examines public normative attitudes toward diverse parenting practices in Japan, with a focus on normative orientations and intergenerational differences. Drawing on survey data from 579 respondents, the results indicate that younger generations are more open to shared parental responsibilities and the involvement of non-family actors, whereas older respondents adhere more strongly to traditional family roles. Attitudes toward the Child Care Commons (CCC)—a framework that envisions shared, network-based child-rearing—were largely supportive, although concerns persisted about parental responsibility and privacy. The findings suggest that value diversification in Japan has begun to reshape ideals of care, yet behavioral and institutional change remain limited. These patterns also suggest that normative differences in child-rearing are intertwined with perceived parenting burdens and role-based expectations, pointing to implications for parental wellbeing under existing institutional constraints. Information and Communication Technologies (ICTs) may help alleviate parenting burdens and privacy challenges, offering pathways toward more inclusive, flexible forms of family support. Achieving such change requires balancing technological innovation with enduring cultural norms—leveraging modern tools to sustain and enrich family relationships in Japan and East Asia.

## 1 Introduction

The environment surrounding children has changed dramatically on a global scale during the 20th and 21st centuries. Behind the declining birthrates that have progressed in many developed countries are changes in people's lifestyles, as exemplified by shifts in the way women work, evolving gender relations, and values. These changes have been described, primarily in the field of sociology, using concepts such as individualization and diversification of the family (Chambers and Gracia, 2021).

Difficulties surrounding child-rearing and childbearing, including the burden of parenting experienced by parents, vary widely from country to country. Previous research has suggested that these difficulties are relatively greater in East Asian countries with marked declines in fertility, and that this is associated with the relative lack of change in East Asian families (Raymo et al., 2015).

Family-related change is often viewed within the theoretical framework of the Second Demographic Transition (SDT) theory in the academic context of sociology and demography (Lesthaeghe, 2014). This theory assumes that in societies that have achieved material wealth, people place more value on higher ideals, such as self-actualization, and it has been posited that changes in societal background, including shifts in values and lifestyles, promote family change in late modern societies (Lesthaeghe, 2014; Lesthaeghe and Surkyn, 2007). Typical outcomes explained by SDT include declining birth rates (Lesthaeghe, 2014; Organisation for Economic Co-operation and Development, 2025a,b), declining marriage rates and an increasing age at first marriage (Sobotka and Toulemon, 2008), the spread of cohabitation (Kiernan, 2001; Thornton and Philipov, 2009), and the increase in births outside of marriage (Organisation for Economic Co-operation and Development, 2022). These family changes have been widely observed in Western societies and have often been cited as evidence supporting the validity of the SDT framework (Lesthaeghe, 2020).

While SDT has explanatory power in elucidating the situation in Western countries (Axinn et al., 2007), it remains unclear to what extent the theory applies to East Asian countries, where social backgrounds, including family values, differ significantly (Lesthaeghe and Surkyn, 2007; Lesthaeghe, 2020). Indeed, East Asian countries are experiencing family changes akin to those in the West, evidenced by the increasing number of people who remain unmarried and the growing prevalence of childless couples. However, East Asian countries, including Japan, deviate from the SDT framework in several aspects: there are significantly fewer children born out of wedlock, cohabitation as an alternative to marriage is not widespread, and the link between marriage and reproduction remains robust (Raymo, 2022; Organisation for Economic Co-operation and Development, 2022; Raymo et al., 2009). Hence, understanding family change in East Asia, particularly in Japan, requires a perspective that extends beyond the SDT framework and situates behavioral differences within specific institutional and cultural contexts.

While East Asian countries share similarities in terms of strong values in family, emphasizing familial responsibility for child-rearing and caregiving, as well as a pronounced decline in fertility, there are also notable country-specific differences (Yasuda et al., 2011; Ishihara and Tabuchi, 2012). Concerning the child-rearing environment, the availability of child-rearing support from extended kinship networks is lower in Japan than in South Korea (Ochiai, 2023). Particularly in Japan, a high degree of the child-rearing burden remains concentrated on mothers (Brinton, 2022; Nukaga and Fujita, 2021), despite some recent progress in terms of institutional support, such as the expansion of childcare centers and the adoption of childcare leave policies.

This suggests that one reason for the challenges in raising children in Japan is the predominance of the “standard” family model, where the mother assumes primary caregiving responsibilities, and there exists limited tolerance for alternative family practices. However, it is unclear how such family values intersect with “non-standard” family practices, such as involving non-family actors in child-rearing, which diverge from conventional family norms. Recent research indicates that gender ideology is a multidimensional construct, with variations across countries along different dimensions (Grunow et al., 2018). Thus, a multidimensional approach is essential for understanding the complex normative attitudes surrounding family and child-rearing. Normative attitudes toward child-rearing are closely intertwined with parental wellbeing, role-based expectations, and the institutional conditions under which caregiving responsibilities are organized and supported.

Building on this perspective, the present study aims to investigate the normative attitudes surrounding child-rearing in Japan, focusing on the dimensions related to the involvement of non-family actors and the distribution of child-rearing responsibilities. In examining these normative attitudes, the study incorporates the concept of the Child Care Commons (CCC) as an analytical framework that situates such attitudes within a broader context of support networks and technological resources. The CCC extends the notion of child-rearing beyond the family by highlighting the potential roles of non-family actors and information and communication technologies (ICT). Using data from a survey of 579 Japanese adults, this study explores (Research Question 1) the extent to which these normative attitudes align—or fail to align—with SDT predictions, and (Research Question 2) whether any divergences may be associated with institutional and cultural constraints. By analyzing both general attitudes toward child-rearing and responses to the CCC concept—an emerging idea not yet embedded in existing family practices—this study seeks to capture the persistence and adaptability of normative attitudes in contemporary Japan.

## 2 Literature review

### 2.1 Empirical evidence of SDT outcomes in Western countries

In Western countries, patterns consistent with the SDT framework—linking value diversification, family practices, and changes in fertility and marriage—have been widely documented by a large body of statistical and empirical evidence.

First, regarding fertility behavior, the total fertility rate has fallen below the replacement level in many countries since the 1970s, with particularly rapid declines observed in Southern and Eastern Europe (Lesthaeghe, 2014). According to OECD Family Database (SF2.1), the total fertility rate in major European countries was observed at around 1.2–1.9 in recent years (Organisation for Economic Co-operation and Development, 2025a,b), a pattern also observed in Japan.

Marriage behavior has also undergone significant changes. The OECD Family Database (SF3.1) shows that marriage rates in many European countries have declined consistently since the 1970s, falling by 30%−50% from 1970s levels by 2020 (Organisation for Economic Co-operation and Development, 2025a,b). The average age at first marriage has risen from the early twenties in the 1970s to around 30 in recent years, indicating a structural delay in the timing of marriage (Sobotka and Toulemon, 2008).

The rise in non-marital births is another typical outcome indicator of SDT. According to the OECD Family Database (Organisation for Economic Co-operation and Development, 2022), in France, the proportion of non-marital births increased from approximately 10% in 1980 to 60% in 2020, while in Sweden and Norway, over half of all births are now to unmarried parents. In recent years, non-marital births account for 40%−60% of all births in about half of European countries. This indicates that in many countries, childbearing outside marriage has become common, largely reflecting the spread of cohabiting unions. However, this phenomenon is not uniform. Perelli-Harris and Gerber (2011), using Russian data, demonstrated that the rise in non-marital births cannot be explained solely by the value shifts assumed by SDT; it is also strongly linked to socioeconomic inequality and insecure living conditions. In other words, while trends broadly consistent with SDT predictions are widely observed across Europe as a whole, the explanatory power of SDT is limited in specific countries and regions. In this context, cohabitation—often institutionalized as an alternative to marriage—is also widely examined as a related pattern of changing family forms. Comparative studies show that cohabitation has become an accepted and stable union type in parts of Northern Europe (Kiernan, 2001), while its diffusion varies substantially across countries depending on institutional contexts and family norms (Sánchez Gassen and Perelli-Harris, 2015).

Synthesizing these statistical and empirical findings, these patterns suggest that in Western societies, value diversification has been consistently reflected in family behaviors, leading to the acceptance of diverse life courses—a process variously described as the diversification or deinstitutionalization of intimate relationships (Cherlin, 2004; Smock and Schwartz, 2020). Furthermore, Lesthaeghe (2020) positions SDT not merely as demographic change but as a restructuring of the value system within Western societies.

### 2.2 Changes in demographic trends in Japan

Changes consistent with SDT theory are evident in indicators of fertility and marriage in Japan. The total fertility rate fell below 2.0 in the mid-1970s and continued to decline thereafter, stabilizing at around 1.3–1.4 from the late 1990s onward. By 2022, it had reached approximately 1.3, one of the lowest levels among OECD countries (Organisation for Economic Co-operation and Development, 2025a,b). Regarding marriage behavior, the marriage rate declined from approximately 10 marriages per 1,000 people in 1970 to 4.1 in 2020 (Statistics Bureau of Japan, 2023), and the average age at first marriage has risen continuously. The proportion of unmarried individuals at age 50 (the so-called lifetime unmarried rate) reached about 28% for men and 18% for women [National Institute of Population and Social Security Research (NIPSSR), 2023], statistically confirming the progression of later marriage and non-marriage. These trends align with the quantitative reduction in births and marriages predicted by SDT theory.

By contrast, indicators related to the diversification of family practices show marked divergence from Western countries. According to the OECD Family Database (Organisation for Economic Co-operation and Development, 2022), although the proportion of non-marital births in Japan has increased slightly in recent years, it remains only around 2%−3% of all births. This is substantially lower than in France and the Nordic countries, where more than half of births are non-marital, indicating that the prevalence of non-marital births has not advanced in Japan.

A similar pattern holds for cohabitation. Raymo et al. (2009), analyzing national survey data, found that while cohabitation in Japan has risen in recent years, most couples transition to marriage within a short period. Thus, cohabitation has not become established as an “alternative to marriage,” as in many Western countries. Furthermore, Mogi et al. (2023), using the recent cohort data, report that while pathways to first marriage, including cohabitation, are diversifying, the linkage between marriage and childbearing remains maintained.

Additional studies noted the social structural factors shaping these trends. Raymo (2003) showed that women's higher educational attainment is associated with delayed first marriage. Raymo and Ono (2007) emphasized the influence of coresidence with parents and economic resources on the transition to first marriage. Retherford et al. (2001) reported that increases in women's labor force participation and major changes in the structure and functioning of the marriage market have contributed to later and fewer marriages in Japan. These findings suggest that while Japan's declining marriage rates and later marriage are closely linked to its social and institutional environment, cohabitation and non-marital childbearing have not become institutionally normalized as they have in Western countries.

In summary, Japanese society exhibits demographic changes consistent with SDT theory, such as declining fertility and marriage rates, yet shows little diversification in family practices such as cohabitation or non-marital childbearing. In other words, Japan possesses a “two-tiered structure”: it fulfills the preconditions of SDT theory, yet some of its typical outcomes have been slow to materialize. This divergence raises theoretical questions about the universality of SDT.

### 2.3 Diversification of values in Japan

In Japan, the diversification of values—a key precondition of the Second Demographic Transition (SDT)—has been suggested primarily by evidence from national social surveys. Although such surveys do not directly measure “value diversification,” they reveal notable shifts in attitudes toward marriage, family, and gender roles over the past three decades. For instance, according to the *National Fertility Survey* conducted by the National Institute of Population and Social Security Research (NIPSSR) (2012, 2023), the share of respondents agreeing that “a couple should have children if they marry” has steadily declined—from 88% in 1992 to 71% in 2010, and further to 55% of men and 37% of women in 2021. This long-term decline indicates a gradual erosion of the once-dominant norm linking marriage and childbearing in Japan.

International data from the *World Values Survey* also provide complementary evidence of gradual change in gender-related values. For example, the share of Japanese respondents agreeing that “being a housewife is just as fulfilling as working for pay” declined from over 70% in the 1990s to around 65% in recent waves, suggesting a slow but discernible weakening of traditional gender-role attitudes (World Values Survey Association, 2022).

Earlier comparative studies (e.g., Raymo et al., 2015) interpreted these developments as part of a broader East Asian pattern, in which rapid socioeconomic transformation has occurred alongside limited change in family norms and institutions. In the Japanese context, however, systematic empirical analysis of value change remains limited—most evidence is descriptive and not designed to capture normative attitudes in a multidimensional framework. As discussed in the following section, this study aims to address this gap by empirically examining normative attitudes toward family and child-rearing in Japan.

### 2.4 Implications from the literature review

Taken together, prior research suggests that Japanese society already meets the preconditions assumed by SDT—such as the diversification of values and rising gender equality—but has not experienced a comparable diversification in family formation. Behavioral changes such as cohabitation and non-marital childbearing remain limited despite clear shifts in values, revealing a persistent gap between value orientation and actual behavior. Scholars have attributed this gap to factors such as the enduring linkage between marriage and childbearing, familistic norms, and institutional constraints. Most of these studies, however, have focused on observable family behaviors—such as marriage, fertility, and cohabitation patterns—rather than on people's normative attitudes. Consequently, little is known about how such attitudes are distributed within Japanese society or how they relate to cultural and institutional contexts. The present study seeks to fill this gap by empirically examining normative attitudes toward child-rearing in Japan. This represents a first step toward clarifying how far SDT's predictions apply to the Japanese case—where they are supported, where they diverge, and how these divergences may be shaped by Japan's specific cultural and institutional conditions.

## 3 Methods

### 3.1 Survey design and question construction

As discussed in the introduction, while value diversification has progressed in Japan, behavioral changes such as cohabitation and non-marital births remain limited. This discrepancy underscores the need to investigate not only overt behaviors but also the underlying structure of normative consciousness. Previous studies have also emphasized that family and gender norms are multidimensional and vary across social contexts (Grunow et al., 2018). Against this background, the survey was designed to capture multiple facets of normative attitudes relating to family practice and child-rearing in a way that would both reflect conventional practices and probe openness to alternative possibilities.

To achieve this aim, we constructed the questionnaire with a progressive three-part structure, each addressing a different thematic focus. The first section, Survey A, consisted of short, structured statements about conventional and emerging child-rearing and family practices, which respondents evaluated on a 7-point scale. This part was intended to capture general attitudes toward familiar practices in a standardized format. The second section, Survey B, was an open-ended questionnaire that asked respondents to describe in their own words what they considered the most important aspects of child-rearing. This format enabled respondents to move beyond fixed categories and articulate personal priorities more freely. The third section, Survey C, introduced the concept of the Child Care Commons (CCC)—a novel and hypothetical institutional framework that respondents were unlikely to have encountered in daily life. It thus provided a future-oriented setting in which participants could reflect on how such unfamiliar arrangements might fit—or conflict—with their existing values and norms surrounding family and child-rearing.

The sequencing was structured to begin with general attitudes, move to personal reflections, and end with a novel institutional proposal, which allowed us to progressively draw out deeper layers of normative consciousness. Moreover, dividing the survey into three parts helped to reduce subjective and cognitive response burden by giving respondents clear thematic boundaries and enabling them to engage more independently with each part, a design principle supported by survey methodology research showing that grouping questions into coherent topical sections improves comprehension and reduces cognitive effort (Dillman et al., 2014; Fowler, 2013). In addition, structuring the three parts so that cognitive demands increased gradually is consistent with principles often noted in survey methodology, which describe that respondents may be more likely to satisfice when cognitive effort is high, and that question order should therefore progress from less demanding to more demanding tasks (Krosnick, 1991; Tourangeau et al., 2000).

By positioning the final section around a hypothetical framework extending beyond current family practices, this structure also enabled us to explore the stability and adaptability of respondents' normative orientations—how firmly they are anchored in present conventions and how flexibly they can accommodate alternative, future-oriented possibilities. This structure thus provided a more reliable way to assess the multifaceted nature of child-rearing norms in Japan, where strong social norms may otherwise constrain the articulation of alternative views.

### 3.2 Contents of the three surveys (A–C)

To capture child-rearing norms in a multifaceted manner, the survey was structured in three parts to progressively engage respondents with general, personal, and novel perspectives on child-rearing. Here, we describe the specific content and response formats of each survey.

#### 3.2.1 Survey A: attitudes toward general statements on child-rearing

**Figure 1:**
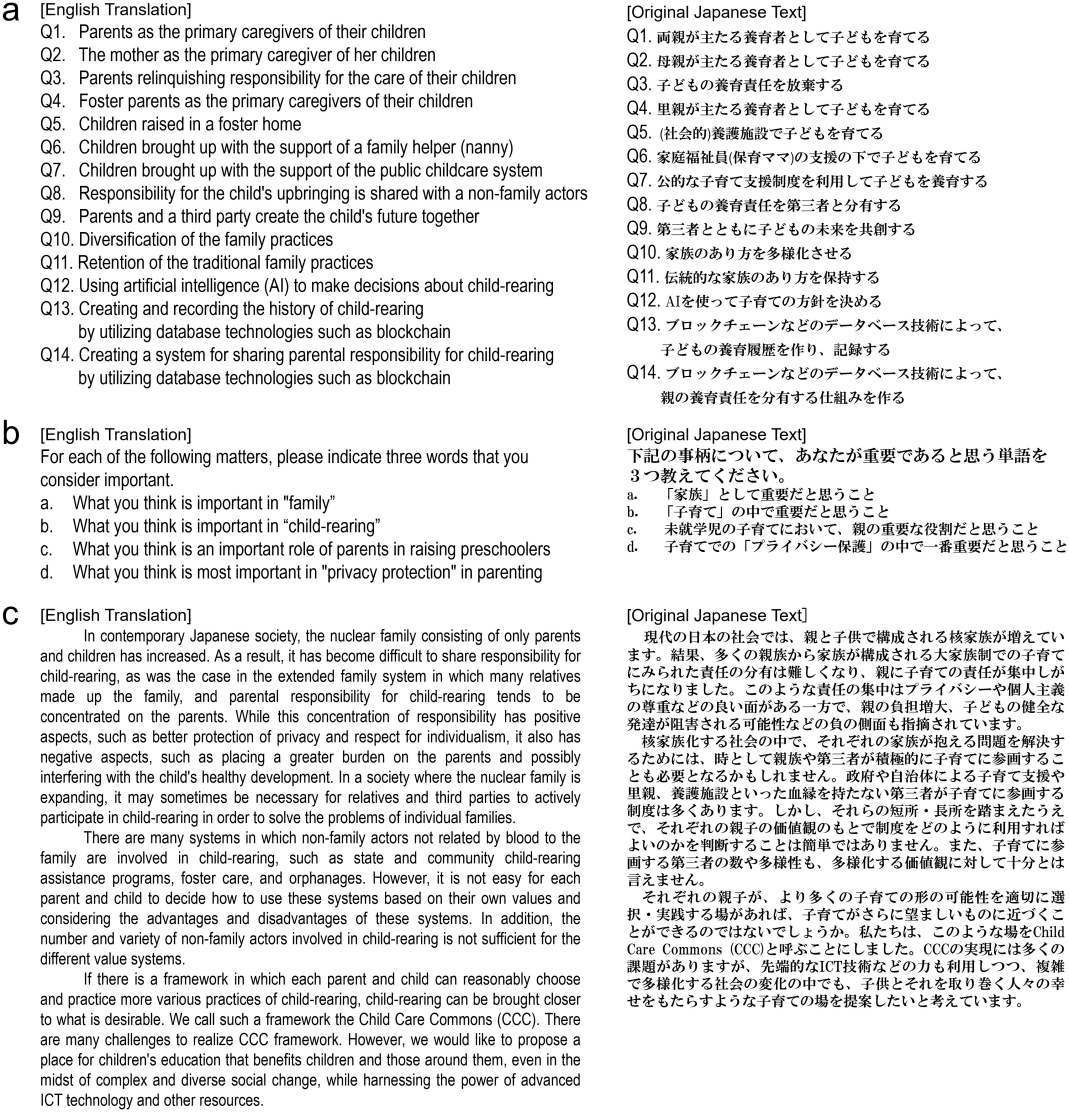
Original text and English translations of Surveys A, B, and C. Only Japanese texts were displayed to the respondents. **(a)** Fourteen questions asked in Survey A. **(b)** The question text used in Survey B. **(c)** The text explaining the Child Care Commons (CCC) system used in Survey C.

Survey A was designed to examine attitudes toward child-rearing practices along an analytical framework that we developed to represent a gradual broadening of social involvement in caregiving. The framework assumes that views on family and child-rearing can be meaningfully organized from the most familiar, family-based arrangements to more external and innovative forms of support. Accordingly, the 14 statements were grouped into four thematic areas (Figure 1a): parents as primary caregivers within the nuclear-family setting (Q1–3), the use of foster care, social care, and public support (Q4–7), the sharing of child-rearing responsibilities with community members or other non-family actors (Q8–9), and possible family practices involving digital or ICT-based collaboration (Q10–14). This progression—from the core family unit to broader and more future-oriented contexts—was intended to capture how acceptance or resistance changes as the imagined sphere of child-rearing extends beyond the conventional household.

Although this framework was inductively constructed for the present study, it aligns with sociological perspectives that conceptualize family change as an expansion of the caregiving domain from the private to the public and communal spheres (e.g., Ochiai, 2023; Chambers and Gracia, 2021). In this sense, the design integrates theoretical insights from prior family research with an empirically grounded structure, making it suitable for comparative analysis within Japan.

Respondents evaluated each statement on a 7-point Likert scale. This module thus provided a structured basis for comparing attitudes toward existing, expanded, and prospective child-rearing practices within the Japanese context.

#### 3.2.2 Survey B: open-ended reflections on important aspects of child-rearing

Survey B invited respondents to describe, in their own words, what they considered most important in child-rearing (Figure 1b). Specifically, they were asked to list three key items under four categories: “*family*,” “*child-rearing*,” “*the role of parents in raising preschool children*,” and “*privacy*.” The open-ended format encouraged respondents to move beyond predefined categories and articulate personal priorities. These responses provide qualitative evidence of how participants conceptualize child-rearing norms, complementing the structured items in Survey A. The open-ended responses were later coded into thematic categories for analysis (see Section 3.6.2).

#### 3.2.3 Survey C: attitudes toward the Child Care Commons (CCC)

Survey C examined respondents' attitudes toward the Child Care Commons (CCC)—a proposed institutional framework that extends child-rearing beyond the standard family model by enabling combinations of diverse resources, including non-family actors and ICT (Figure 1c). After reading a standardized description of CCC, respondents first indicated their agreement on a 7-point scale and then provided a brief written rationale of approximately 100 words. Finally, to reflect the fact that CCC envisions participation by multiple types of actors rather than only parents, respondents were asked to evaluate their willingness to participate under four imagined roles (*parent, child, friend*/*acquaintance*, and *third party*) on a 5-point scale. Because CCC is largely unfamiliar to the general public, respondents' evaluations can be interpreted as reflecting how existing family norms shape acceptance or resistance to new proposals. In this sense, Survey C provides a useful perspective on both the constraints and the potential flexibility of normative consciousness.

### 3.3 Procedure and ethical considerations

A custom-made survey system provided by NTT Communications Online Marketing Solutions and operated by NTT DATA Management Research Institute was used for recruitment, response, and data collection. Registrants accessed a designated survey website using their own device (PC/smartphone, etc.) and answered the questionnaire. On the accessed page, an informed consent form was displayed prior to the survey, and only those who agreed to the form were allowed to proceed to the next page of the survey. The informed consent and the implementation of the questionnaire were reviewed and approved in advance by the Ethics Review Committee of NTT Communication Science Laboratories.

### 3.4 Sampling and recruitment

We contracted a research firm (NTT DATA Management Research Institute) to conduct an online survey. Respondents were recruited from January 27 to February 7, 2023, targeting 2,854 persons from the firm's Internet panel, using quota sampling to balance gender and age categories. Approximately 100–130 respondents were targeted for each age group (20s, 30s, 40s, 50s, and 60s+). This procedure yielded 579 valid responses for analysis (see Section 3.5 for demographic details).

Although the survey was not based on random sampling, the quota design ensured roughly equal numbers across gender and age groups, prioritizing comparability between demographic subgroups over full population representativeness. Because all participants were drawn from the same Internet panel under common recruitment conditions, we consider the risk of systematic bias in generational or gender-based comparisons to be limited. Nevertheless, caution is warranted when generalizing the findings to the entire Japanese population, a limitation we revisit in the Discussion section.

These registrants were general members of the public residing in various prefectures throughout Japan. They had registered themselves as monitors to answer various questionnaires conducted by research firms, including those not included in this study. Upon completion of the surveys, registrants were awarded points in accordance with the research firm's criteria, which could be redeemed later for various forms of reward. Although the firm's internal criteria were not disclosed, respondents were aware of the rewards they would receive for participating in the survey before responding.

### 3.5 Respondent characteristics

**Figure 2:**
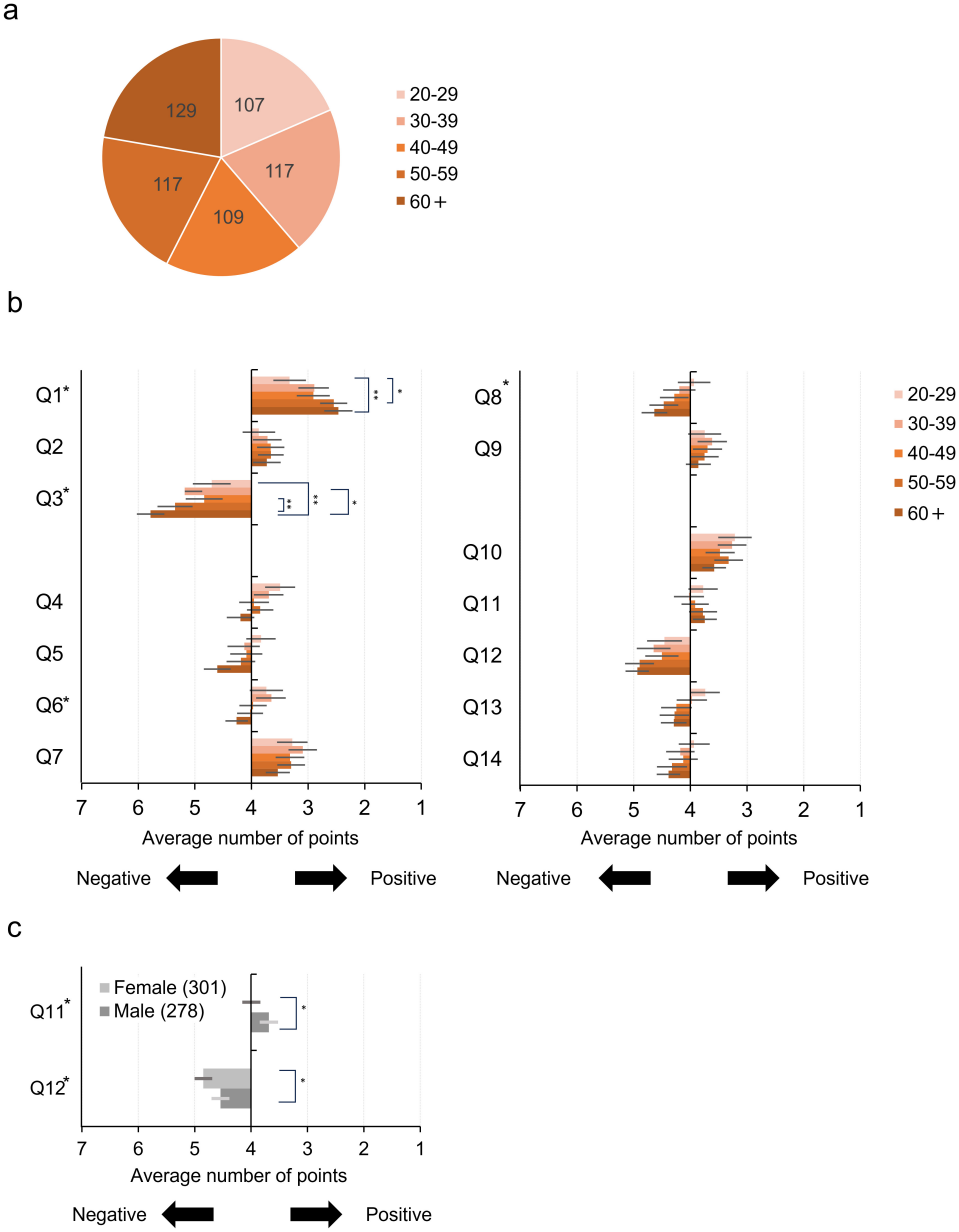
Results of Survey A. **(a)** Age distribution of all respondents. **(b)** Mean rating of each question by age group. **(c)** Mean ratings of Q11 and 12 by gender. In panels **(b)** and **(c)**, error bars show 95% confidence intervals. **denotes *p* < 0.01, *denotes *p* < 0.05.

A total of 579 valid responses were obtained (278 men, 301 women; age: *M* = 45.6, SD = 13.8; range: 20–70 years). Figure 2a shows that the age distribution was broadly balanced across decades, with each age group (20s, 30s, 40s, 50s, and 60s+) represented by roughly 100–130 individuals.

Respondents resided in 44 prefectures, with distributions somewhat skewed toward densely populated areas: Kanto (270), Kansai (106), and Chubu (80), followed by Kyushu/Okinawa (43), Hokkaido/Tohoku (45), and Chugoku/Shikoku (35). Although regional representation was not fully proportional to population size, the sample covered all major regions of Japan.

The respondents were non-specialists who had voluntarily registered as survey monitors with the contracted research firm. Their participation was incentivized by a point-based reward system, in line with standard practices for online research panels. While such panels may differ in some respects from the general population, the deliberate use of quotas resulted in broadly equal numbers across age and gender groups, supporting the reliability of demographic comparisons in this study.

### 3.6 Indicators and statistical analysis

Our analytic strategy combined quantitative and qualitative approaches to capture the multifaceted nature of child-rearing norms while enabling transparent demographic group comparisons. Likert-type ratings were analyzed using robust non-parametric factorial tests based on the aligned rank transform (ART-ANOVA), which permit interpretable tests of main and interaction effects without strong distributional assumptions. Free-text responses were systematically coded into thematic categories to provide qualitative evidence complementary to the structured items. The indicators derived from each survey, along with the corresponding analytic procedures, are detailed below.

#### 3.6.1 Indicators from Survey A (general attitudes) and analysis

Survey A collected responses to 14 short statements on a 7-point Likert scale. Items were designed to span four thematic domains introduced in Section 2.3: (a) parents as (primary) caregivers (Q1–Q3); (b) use of foster/public/social care (Q4–Q7); (c) sharing responsibility with third parties (Q8–Q9); and (d) possible family practices and the use of ICT (Q10–Q14).

Analytically, we treated the items as ordinal and conducted item-level comparisons (domain labels are used to aid interpretation, but tests are run per item). We used ART-ANOVA to analyze the data in Survey A (Wobbrock et al., 2011; see Section 3.6.4 for details). We applied the aligned rank transform (ART) procedure, followed by three-way factorial ANOVA with age group (20s, 30s, 40s, 50s, 60s+), gender (male, female), and parental status (with/without children) as between-subject factors. Where main or interaction effects were significant, we performed *post-hoc* multiple comparisons using ART-C (Elkin et al., 2021).

#### 3.6.2 Indicators from Survey B (open-ended reflections) and analysis

Survey B asked respondents, in their own words, to list three key items under each of four domains: family, parenting, role of parents in raising preschool children, and privacy. The emphasis in this survey was on identifying key thematic priorities and describing how their relative salience differed across demographic groups, rather than on testing specific statistical hypotheses.

Responses were first segmented into individual lexical units and then coded into thematic categories. One of the authors independently grouped semantically similar responses. A second author cross-checked the mapping against the originals, and discrepancies were resolved through discussion between the authors to ensure reliability.

To enable systematic comparison, we computed the relative frequencies of each category across the four domains. These distributions were then cross-tabulated by age group, gender, and parental status. While the primary focus was descriptive, this approach allowed us to identify whether certain themes appeared more prominently in specific demographic groups, complementing the structured attitudinal measures from Survey A.

In addition, to examine whether the distribution of thematic categories significantly differed across demographic subgroups, omnibus chi-square tests were performed for each domain (family, parenting, role of parents, and privacy). Adjusted residuals were then inspected to identify categories showing notable deviations from expected frequencies, and false discovery rate (FDR) correction was applied to control for multiple comparisons.

#### 3.6.3 Indicators from Survey C (attitudes toward CCC) and analysis

Survey C evaluated the Child Care Commons (CCC) concept on three indicators: (i) agreement with CCC on a 7-point Likert scale after reading a standardized description; (ii) a ~100-word written rationale explaining the rating; and (iii) willingness to participate on a 5-point scale under four imagined roles (parent, child, friend/acquaintance, third party). Agreement ratings were analyzed using ART-ANOVA (see Section 3.6.4) with the same three between-subject factors as in Survey A. Written rationales were thematically coded following the Survey B procedure, and role-based willingness ratings were summarized descriptively across roles and demographic subgroups.

#### 3.6.4 Rationale for the factorial ART-ANOVA approach

Several analytic strategies could be considered for this dataset, including regression-based models. Regression is highly flexible and allows covariates to be included, but the number of estimated parameters increases rapidly once categorical predictors with multiple levels and their interactions are modeled. This expansion requires relatively large sample sizes for stable estimation (Green, 1991), which can compromise the interpretability of present findings given our focus on factorial group comparisons.

In contrast, factorial ANOVA is particularly suitable for designs with a limited set of categorical factors. According to power analysis guidelines (Cohen, 1988), detecting a medium-sized effect (*f* ≈ 0.25) at 80% power typically requires ≈20 observations per cell. With *N* = 579 distributed across 20 cells (5 age groups × 2 genders × 2 parental statuses), our design yielded approximately 29 observations per cell, exceeding the threshold. This supports adequate power not only for main effects but also for interaction effects, which are central to our research question.

We therefore adopted the aligned rank transform (ART) ANOVA (Wobbrock et al., 2011) for Likert-scale outcomes in Surveys A and C. The ART-ANOVA combines the interpretability of factorial ANOVA with the robustness of non-parametric methods, making it well-suited for ordinal survey data that may not meet normality assumptions. When significant effects were identified, *post-hoc* tests were conducted using ART-C (Elkin et al., 2021).

This mixed-methods strategy—quantitative analysis of Likert-type data via ART-ANOVA and qualitative categorization of free-text responses—provides a robust and interpretable basis for demographic group comparisons, aligning closely with the study's aim.

## 4 Results

To systematically summarize the results, we organized the main empirical patterns according to the two central questions of this study. The detailed findings, including statistical analyses, are presented in the subsequent sections.

RQ1: To what extent do normative attitudes in Japan correspond to or deviate from SDT-related expectations?

Both Survey A and Survey B revealed a consistent cross-generational pattern. Younger adults exhibited attitudes more closely aligned with SDT-related predictions, expressing greater openness to the sharing of parental roles and to the involvement of non-family actors. In Survey A, this was reflected in more favorable evaluations of role-sharing items (e.g., Q6, Q8), whereas older adults expressed stronger support for exclusive parental responsibility (e.g., Q1, Q3). Survey B confirmed this contrast: younger individuals more frequently emphasized concrete burdens—time, money, and effort—while older individuals highlighted emotional and relational values such as affection, trust, and discipline. Taken together, these complementary findings indicate partial alignment with SDT-consistent orientations among younger adults and a persistent adherence to traditional expectations among older adults.

RQ2: How are these differences shaped by institutional and cultural constraints?

The limits to acceptance suggested by Surveys A and B—particularly norms emphasizing parental responsibility, emotional bonds, and privacy—were further clarified in Survey C. Although a clear majority of respondents across all age groups evaluated the Child Care Commons (CCC) concept positively, their open-ended responses repeatedly expressed concerns about parental responsibility, privacy protection, and the involvement of non-family actors. These concerns were widespread across demographic groups, suggesting that culturally rooted expectations are broadly applicable rather than confined to particular generations. Furthermore, willingness to participate in the CCC was more divided than evaluations of the concept itself, indicating that such expectations influence perceptions of the feasibility of adopting new institutional arrangements.

These findings show the points of correspondence and divergence with SDT-related expectations and identify the cultural factors underlying these differences. These results provide the empirical basis for the interpretations developed in the Discussion section.

### 4.1 Survey A

The results of Survey A are shown in Figure 2. The results showed that the main effects of age for Q1, Q3, Q6 and Q8 (Q1: *F* (4, 559) = 4.01, *p* < 0.005, Q3: *F* (4, 559) = 5.47, *p* < 0.0005, Q6: *F* (4, 559) = 4.01, *p* < 0.005, Q8: *F* (4, 559) = 2.68, *p* < 0.05) and the main effect of gender for Q11 and Q12 (Q11: *F* (1, 559) = 5.80, *p* < 0.05; Q12: *F* (1, 559) = 5.57, *p* < 0.05), and the interaction between gender, age, and having children in Q4 were significant (*F* (4, 559) = 4.0, *p* < 0.005).

The main effects of age (Q1, Q3, Q6 and Q8) and the interaction (Q4) were subjected to multiple comparisons using ART-C (Elkin et al., 2021). The results are summarized in Table 1. In Q1, a significant difference was observed between the group aged 20–29 and the groups aged 50–59 and 60s+. In Q3, significant differences were observed between the group aged 60s+ and the groups 20s,30s,40s. In Q6, a significant difference was observed between the groups aged 30–39 and 60s+.

**Table 1:** Results of multiple comparisons of statistical analysis for data of Survey A.

**Question number**	**Contrast**	**Estimate**	**SE**	**df**	***t*.ratio**	***p*-value**
Q1	B1–B4	92.94	28.91	559	3.21	0.012^*^
	B1–B5	102.45	29.97	559	3.42	0.007^**^
Q3	B1–B5	−112.83	29.76	559	−3.79	0.001^**^
	B2–B5	−67.36	23.58	559	−2.86	0.036^*^
	B3–B5	−89.21	23.28	559	−3.83	0.001^**^
Q6	B2–B5	−77.14	23.73	559	−3.25	0.012^*^
Q4	Male: 60s+: chidren—female: 20–29: non-children	144.20	32.67	559	4.41	0.002^**^

B1: 20–29, B2: 30–39, B3: 40–49, B4: 50–59, B5: 60s+. ^**^denotes *p* < 0.01, ^*^denotes *p* < 0.05.

The results indicated that with increasing age, respondents were more resistant to the involvement of third parties. Significant differences in Q1, Q3, Q6 and Q8 (Figure 2b) consistently indicated that younger generations have more positive attitudes toward others to share the parental role. Questions Q1 and Q3 examine attitudes toward parental responsibility and its relinquishment. As the respondents' age increases, their attitude toward parental care becomes more positive (Q1) and more negative toward relinquishing parental responsibility (Q3). In other words, these results suggest that as respondents age, their emphasis on parental responsibility becomes stronger. Q6 and Q8 examined the attitudes toward support for child-rearing by non-family actors (including family helpers).

Although the range of these generational differences is relatively small, both attitudes consistently become more negative with increasing age, indicating greater resistance to the involvement of third parties. These questions consistently indicate that the older respondents are, the more strongly they emphasize the importance of the role of parents and the greater their resistance to role substitution by non-family actors. However, it should be noted that the magnitude of the increase was not very large. While the effect of age on the perception of non-family actors' participation in child-rearing is consistent, its effect size appears to be limited, at least among our respondents.

Additionally, Q11 and Q12 assessed attitudes toward traditional family forms and the integration of artificial intelligence (AI) into family decision-making. In responses to these questions, modest differences were found depending on the respondents' gender (Figure 2c). Specifically, men indicated slightly more positive attitudes than women toward traditional family forms and the integration of AI. The differences in responses to these two questions may reflect a tendency among some male respondents to express relatively greater openness to technological solutions, as opposed to sharing responsibilities with non-family actors, as a way of maintaining traditional family forms. However, given the small differences observed in the current results, it is best to assume that gender differences are not particularly large.

### 4.2 Survey B

**Figure 3:**
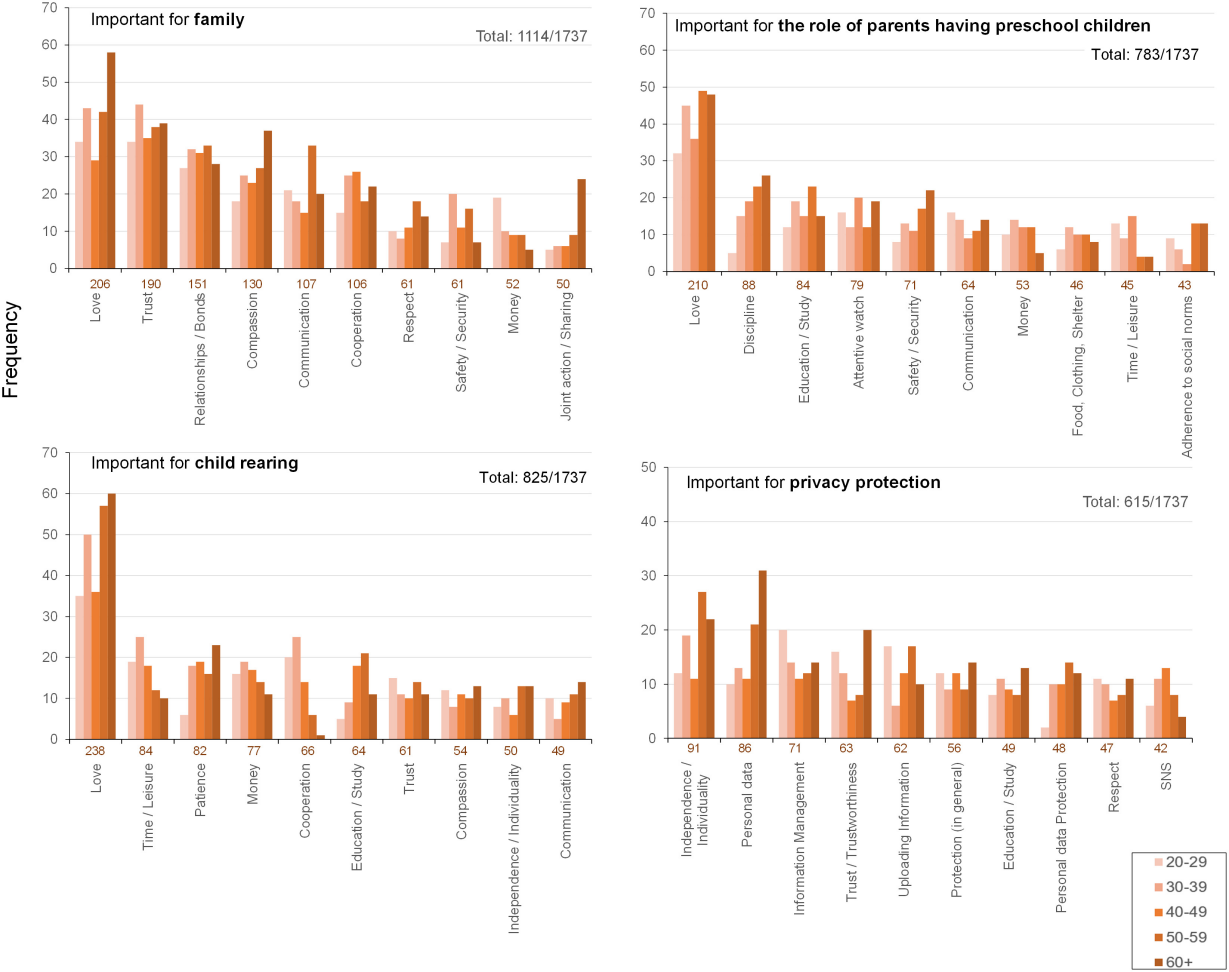
Results of Survey B. Each box shows the words that rank in the top 10 in frequency of occurrence out of a total of 1,757 words mentioned by all respondents in four word categories. The numbers in the upper-right corner of each graph indicate the total frequency of words in the top 10.

Figure 3 shows the frequency distributions of word categories for each of the four open-ended questions. In the question about what is important to the family, human-relationship-related categories such as love, trust, bonding, and compassion were most frequently expressed, followed by communication, cooperation, and respect. For child-rearing, love was again the most common response, followed by categories reflecting material conditions such as time, patience, and money. For the role of parents of preschoolers, love was the most frequently mentioned aspect, followed by categories related to education such as discipline, education, and childcare. Finally, for the question of what is important for privacy protection, personal independence was the top category, followed by categories related to digital data management, such as personal data, data management, and trust.

Chi-square omnibus tests were then conducted to examine whether the distribution of categories differed by age group. Results indicated significant heterogeneity only in the family domain (χ^2^ (760) = 827.14, *p* = 0.045, Cramer's *V* = 0.35). For child-rearing, the omnibus test did not reach significance (χ^2^ (920) = 969.91, *p* = 0.12), and for the role of parents, no age differences were detected (χ^2^ (1036) = 1,069.50, n.s.). In the privacy domain, results showed a weak trend toward significance (χ^2^ (1532) = 1,614.76, *p* = 0.069, Cramer's *V* = 0.48), suggesting that generational variation in how independence and information management are framed may exist, although the statistical evidence remains inconclusive. However, when residuals were examined, no single category difference reached a significant level after FDR correction for multiple comparisons.

Adjusted residuals were further examined to explore distributional tendencies underlying the omnibus results (Table 2). Several categories showed residuals exceeding |2|, indicating trend-level deviations from expected frequencies. Mentions of “Money” were markedly more frequent among respondents in their twenties (adjusted residual = +3.4; 19 cases) and less common among those aged sixties or older (−2.2; five cases). A similar cross-generational contrast appeared for “Safety/security,” which was emphasized by participants in their thirties (+2.5; 20 cases) but seldom noted among the oldest group (−2.1; seven cases). In contrast, relational and emotional themes tended to be more salient among older respondents: “Love” (+2.2; 58 cases; 60s+), “Joint activities” (+4.4; 24 cases; 60s+), and “Watching over” (+3.1; four cases; 60s+) showed positive deviations. Middle-aged respondents in their fifties showed a positive deviation for “Communication” (+2.8; 33 cases).

**Table 2:** Results of residual analysis in chi-square analysis for data of Survey B.

	**Age**					
**Response category**	**20–29**	**30–39**	**40–49**	**50–59**	**60s+**	**Total**
Love	−0.8 (34)	0.3 (43)	−1.9 (29)	0.1 (42)	**2.2 (58)**	206
Communication	0.3 (21)	−0.9 (18)	−1.3 (15)	**2.8 (33)**	−0.9 (20)	107
Safety/security	−1.4 (7)	**2.5 (20)**	−0.2 (11)	1.2 (16)	**−2.1 (7)**	61
Money	**3.4 (19)**	−0.2 (10)	−0.3 (9)	−0.5 (9)	**−2.2 (5)**	52
Joint activities	−1.6 (5)	−1.5 (6)	−1.3 (6)	−0.4 (9)	**4.4 (24)**	50
Kindness	**2.6 (11)**	−0.9 (4)	0.6 (7)	−1.9 (2)	−0.3 (6)	30
Family lineage	0.3 (2)	**2.6 (5)**	−0.6 (1)	−1.5 (0)	−0.8 (1)	9
Home	−1.2 (0)	**2.8 (4)**	0.9 (2)	−1.2 (0)	−1.3 (0)	6
Homebase	−1.2 (0)	−1.2 (0)	**2.0 (3)**	1.8 (3)	−1.3 (0)	6
Watching over	−1.1 (0)	−1.1 (0)	−1.1 (0)	0.0 (1)	**3.1 (4)**	5

All residuals are shown prior to FDR correction, and no category remained significant after correction (*p* > 0.05). Values are adjusted residuals; counts are shown in parentheses. Bold indicate residuals exceeding |2|.

Mentions of “Home” (+2.8; four cases; 30s) were most frequent among respondents in their thirties, whereas “Homebase” (+2.0; three cases; 40s; +1.8; three cases; 50s) increased among middle-aged respondents. “Family lineage” (+2.6; five cases; 30s) showed a similar mid-life concentration, while “Kindness” (+2.6; 11 cases; 20s) was more frequent among younger participants. These patterns may suggest a gradual life-course transition from building to sustaining the home as the relational center of family life. In addition to the categories meeting the |2| threshold in Table 2, near-threshold tendencies also appeared. “Peace” showed a positive deviation in the twenties (e.g., +1.9; eight cases) with lower values in older groups (−0.9; three cases; 50s / −1.2; three cases; 60s+), and “Responsibility” trended positive among the oldest respondents (+1.7; 14 cases; 60s+). These did not meet the inclusion criteria for Table 2 but are documented in Supplement 1 (Table S1), which lists residuals for all categories with total frequency ≥5. Although none of these contrasts remained significant after the FDR correction, the residual pattern as a whole indicates age-related shifts that are best interpreted as exploratory.

Taken together, the results in Survey B showed that while “Love” and “Trust” were broadly shared across all generations, the relative weight given to material, structural, and psychological resources shifted with age. Younger respondents emphasized material burdens such as “Money” (and related structural concerns such as “Home” in formation), whereas middle-aged respondents stressed “Communication,” “Homebase,” and “Family lineage,” reflecting transitional attention to relational stability. Older respondents, in turn, highlighted “Love,” “Joint activities,” and “Watching over,” suggesting a shift toward maintaining emotional connection and intergenerational continuity rather than managing material or structural conditions. At the same time, “Kindness” (and, at a near-threshold level, “Peace”) appeared more often among younger participants, whereas “Responsibility” trended higher in older groups (see Table S1 for details). The prominence of material and structural concerns among younger respondents illuminates the perceived burdens of child-rearing in early adulthood, whereas the greater emphasis on relational and normative qualities in older groups aligns with stronger intergenerational responsibility. These generational contrasts provide a contextual basis for interpreting the structured findings of Surveys A and C, suggesting that acceptance or resistance to non-family actors and ICTs is grounded in the material conditions and relational norms identified in Survey B.

### 4.3 Survey C

**Figure 4:**
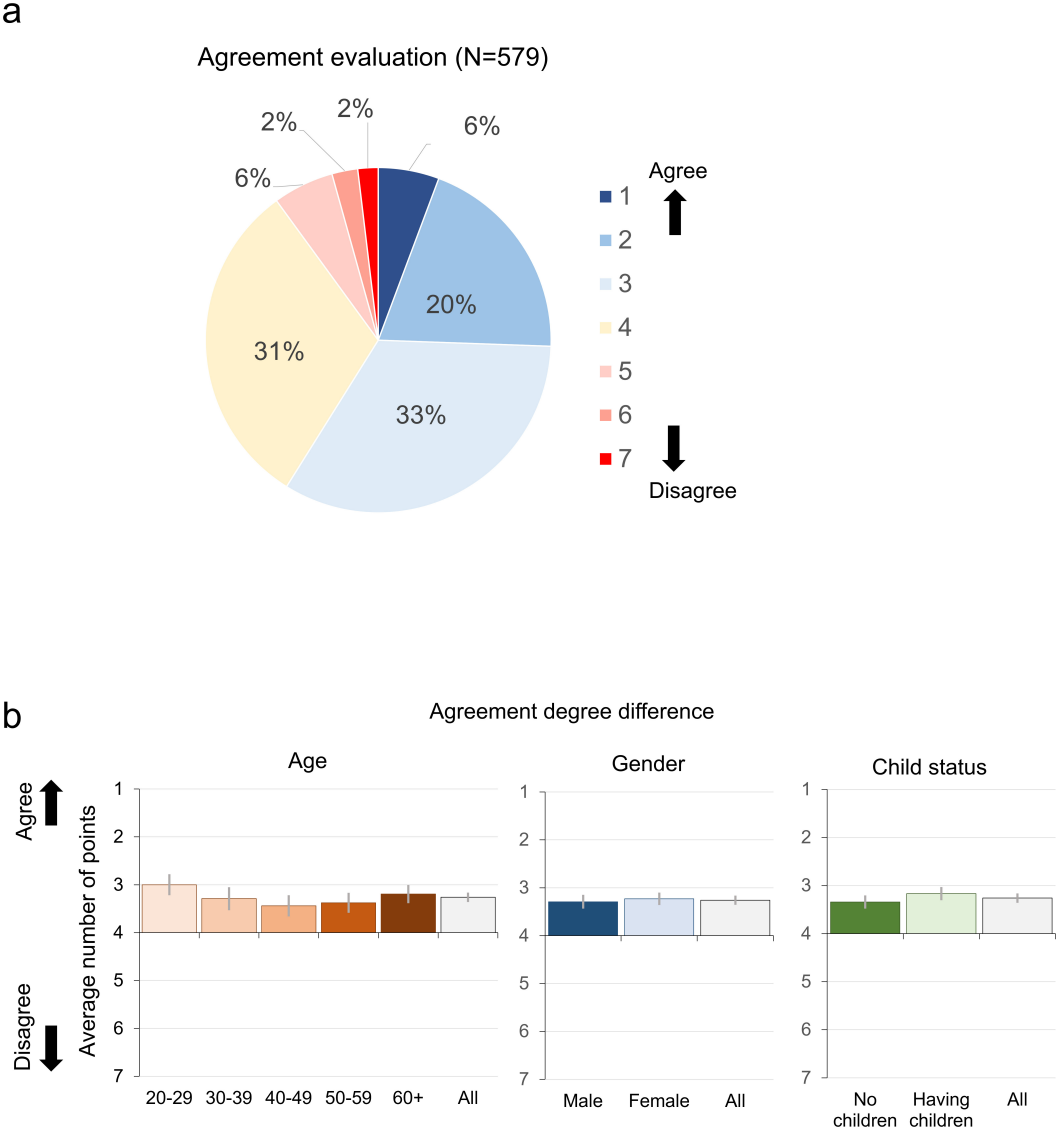
Degree of agreement with CCC. **(a)** Overall results. **(b)** Results for each age, gender, and child status group. Error bars show 95% confidence intervals.

The results of Survey C are shown in Figure 4. Overall, respondents rated the concept of CCC favorably. Approximately 60% of respondents expressed agreement with the concept of CCC, which supports family diversification, including the possibility of non-family actors' involvement. In contrast, about 10% of the respondents indicated some disagreement with CCC (Figure 4a). There was no clear difference between groups divided by gender, age, and having children (*F* (1, 559) = 0.16, n.s. for gender, *F* (4, 559) = 1.92, n.s. for age, *F* (1, 559) = 2.09, n.s. for having children, Figure 4b).

We examined the open-ended responses of those who indicated some disagreement (scores of 5 or higher). One author merged comments from 58 respondents who indicated disagreement into seven categories based on the similarity of comments. Another author cross-checked the original and categorized the data to ensure that the aggregation was carried out appropriately. Among the responses, as shown in Table 3, there were many comments expressing concerns about parents abdicating their responsibilities (13 comments were categorized as “Emphasize the responsibility of the parents”). In addition, there were many concerns about resistance to non-family actors' participation itself (10 comments), as well as about invasion of privacy and loss of flexibility in family form (10 comments).

**Table 3:** Reasons given by those who had a high level of disagreement (5 or more).

**Comments from respondents answered “disagree”**		
**Comments category**	**Frequency**	**Comment examples**
Emphasize the responsibility of the parents	13	•Parents should be responsible for their children •Concentration of responsibility on the parents is inevitable since they gave birth to the child •Parents need to take maximum responsibility for their child rearing
Disagreement with third-party participation	10	•It seems impossible for a third party to take charge of parenting on behalf of the parents •It is not clear who can guarantee the safety of the child with the involvement of a third party •(The third party) is not in a position to take or be able to take responsibility in the event of an emergency
Invasion of privacy and independence	10	•It is an invasion of privacy protection •What is desirable or best is case by case •Parents have their own values and only those who need to be involved should be able to have a third party participate
Lack of feasibility	5	•I understand the idea, but it's hard to accept in reality •I feel it's all ideals and it's hard to realize
Emphasize the preservation of the traditional family	4	•As much as possible, the family should take the traditional form of the family •I think the nuclear family is the modern form. It's not all that bad
Doubts about the effectiveness of ICT implementation	4	•I doubt that the power of ICT technology is necessary •Not cost-effective
Other comments	12	•(This idea) does not lead to financial support •Need help from the government •Somehow (this idea) didn't resonate with me

To illustrate these tendencies, representative written remarks from respondents who disagreed with the CCC included (Table 3): one respondent (CCC rating = 7) wrote, “Parents should bear the utmost responsibility for child-rearing,” another (rating = 7) noted, “After all, non-family are still outsiders; it is better not to involve them,” and a third (rating = 7) commented, “There is no need for third parties to intervene—it would only increase risks and violate privacy.” These comments exemplify how emotional and moral expectations surrounding parental roles underlie negative perceptions of the CCC concept.

Although similar terms such as “parental responsibility” or “privacy” occasionally appeared among those who were generally supportive of the CCC (ratings 1–3), their meanings differed. For example, one supportive respondent (rating = 2) stated, “If parents bear all responsibility for child-rearing, the stress becomes overwhelming, so external support is essential,” and another (rating = 3) wrote, “Society as a whole should share in child-rearing, and third-party involvement is important.” These remarks suggest that while respondents across groups recognized similar issues—such as the heavy burden on parents and the need for social support—they differed in how these problems should be addressed. In other words, similar vocabulary can express contrasting orientations depending on whether it is used to defend traditional roles or to propose shared responsibilities.

**Figure 5:**
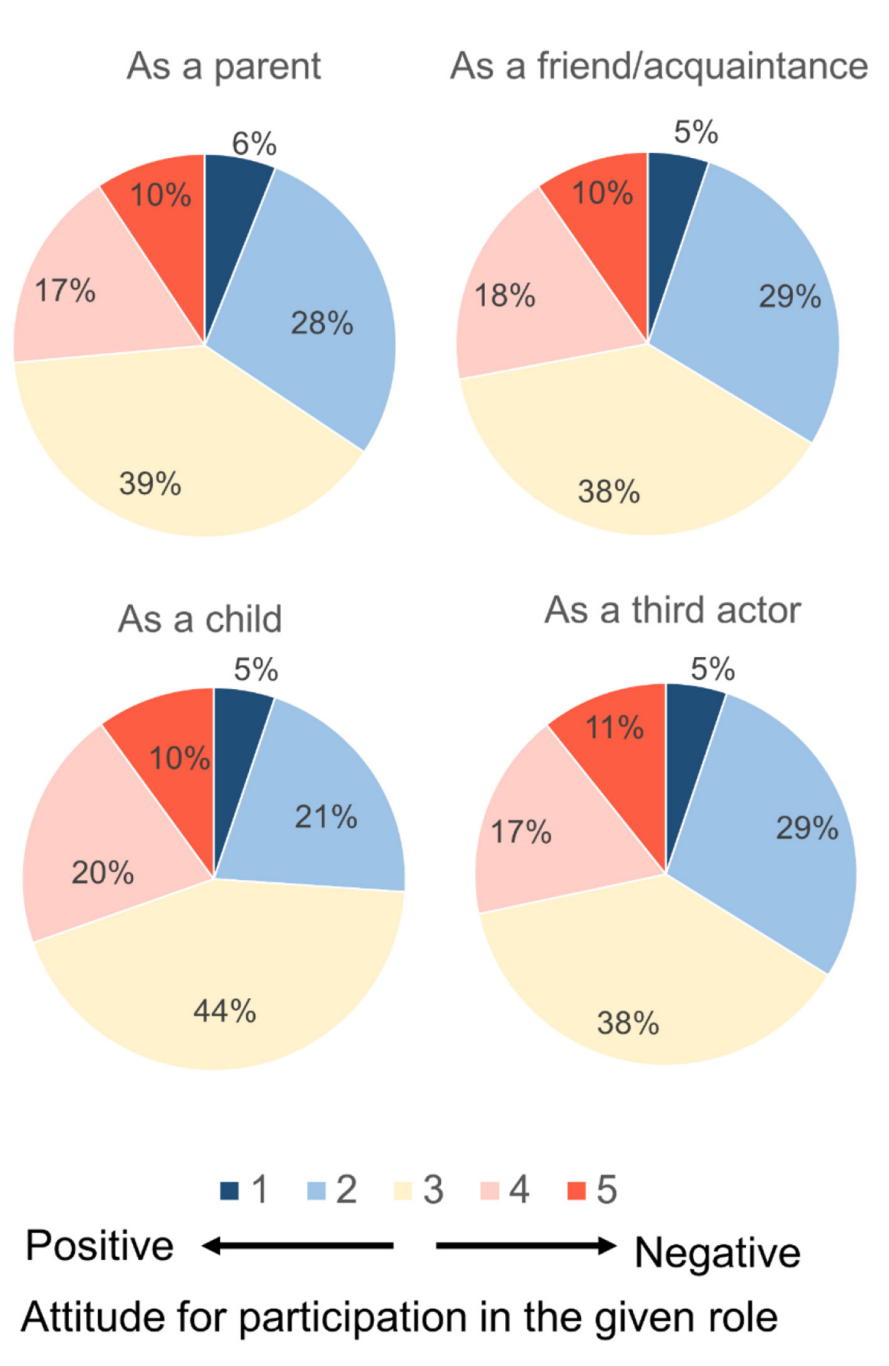
Attitudes toward participating in the CCC system in a given role.

The results of the item assessing respondents' attitudes to participate in the CCC system from the perspective of an assigned role revealed no significant variation across the roles of parent, friend, and third party (Figure 5). Approximately one-third of participants indicated a positive attitude toward participation (i.e., selecting 1 or 2 on a 5-point scale), around 40% responded neutrally (3), and the remaining third expressed a negative attitude (4 or 5). In contrast, when respondents considered the perspective of the child, the proportion expressing willingness to participate declined to 26%, with 44% selecting a neutral response and 30% indicating a negative stance.

Notably, the proportion of participants expressing a positive attitude toward actual participation was lower than approximately 60% who had previously approved of the system from an objective standpoint. Nonetheless, the fact that roughly 30% responded positively suggests that the CCC system could be regarded as a partially acceptable option. The decline in positive responses when adopting the child's perspective warrants particular attention.

Given that all participants in the present survey were adults—many of whom were likely raised within traditional parental care frameworks—it is plausible that a degree of discomfort was elicited by childcare environments that differ from their own upbringing. To gain a more comprehensive understanding of societal attitudes toward the CCC system, further investigation involving younger populations, including minors, would be required.

## 5 Discussion

### 5.1 Consistency and divergence from SDT predictions

The primary objective of this study was to examine the normative attitudes of the Japanese public toward diverse child-rearing practices based on the Second Demographic Transition (SDT) and the newly introduced concept of the *Child Care Commons* (CCC). In relation to Research Question 1, the results of the three surveys revealed both consistencies and divergences from the expectations of SDT.

On the one hand, attitudes toward the involvement of non-family actors in child-rearing were generally positive (Survey A), with only about 10% of respondents expressing disagreement with the introduction of the CCC, which suggests that raising children can involve networks of people beyond the household (Survey C). Younger respondents, in particular, showed greater openness to sharing parental roles and to the participation of non-family actors in child-rearing, and a majority of participants endorsed the concept of the CCC. These findings suggest that certain normative shifts are broadly consistent with the predictions of SDT, even within a cultural context characterized by strong traditional family norms, such as Japan.

At the same time, the persistence of strong beliefs in parental responsibility reveals enduring cultural and institutional barriers. The results of Survey B showed that *love* and *trust* were frequently mentioned as important factors in family and parenting across all age groups. Additionally, *personal independence* and *information management* were also considered important for protecting privacy. Affection and trust are often perceived as difficult to extend to third parties, and the involvement of non-family actors is seen as potentially compromising individuals' ability to safeguard their privacy or ensure that their preferences and values are respected. Comments in Survey C (Table 3) further revealed concerns about the possible erosion of traditions, cultural values, and parental responsibility. Some respondents feared that encouraging non-family participation might lead to an abdication of parental duties and negatively affect children's development.

Taken together, these results suggest that cultural factors, including the continuing heavy burden on mothers and the persistence of the “standard family model,” help explain why value diversification in Japan does not easily translate into behavioral change. While the surveys cannot establish causality, they suggest that Japanese child-rearing norms are evolving at the level of ideals; however, this has not automatically led to corresponding behavioral transformations.

Importantly, the present findings suggest that, at least among younger generations, there is a shift in attitudes toward parental responsibility. Younger respondents placed greater emphasis on material constraints such as time, financial resources, and external support, and showed less insistence on strict adherence to conventional expectations of parental roles. This pattern may reflect a pragmatic adjustment of attitudes under physical and economic limitations. In this sense, reduced insistence on strictly upholding conventional expectations may function as a coping strategy in response to constrained parenting conditions, potentially helping to mitigate declines in parental wellbeing associated with material and time-related pressures.

The findings also suggest the relevance of differentiated parenthood trajectories, understood here not as divergent family forms but as differences in life-course stage and imagined parental roles. Responses in Survey C varied depending on whether participants considered the perspective of a parent, a child, or a third party, indicating that evaluations of shared parental responsibility and diversified child-rearing arrangements were not uniform across assumed roles.

These role-based differences suggest that expectations regarding responsibility sharing are considered from positions associated with different levels of anticipated involvement and burden. Such positions are not independent of the practical conditions under which families operate, including the time, economic resources, and childcare support available to them. In this sense, the observed attitudinal differences can be interpreted in relation to differences in access to time, economic resources, and childcare support, which are closely related to broader issues of social inequality and welfare provision. Further research using more detailed empirical approaches would help to clarify these relationships.

### 5.2 Rethinking SDT in the East Asian context

The results in this study invite a reconsideration of the original SDT theory developed based on Western European experiences. The theory posits that declining fertility and marriage rates were accompanied by the institutionalization of alternative family forms, including cohabitation and non-marital childbearing. In those contexts, changes in values and lifestyles were supported by adaptive policy frameworks—gender-equal welfare systems, family law reforms, and public childcare—that legitimized new patterns of family life. Meanwhile, the persistence of the “standard family model” in Japan functions as a mediating structure that absorbs cultural change without allowing it to reconfigure everyday practices. This indicates the importance of contextualizing SDT not as a universal sequence, but as a process contingent on local configurations.

Japan and other East Asian societies appear to exhibit a mismatch between values and cultural structures that remain largely unchanged. This is interpreted as asymmetric value diversification without corresponding behavioral pluralization. A similar pattern can be observed across other East Asian societies, where rapid modernization has not necessarily led to the diversification of family forms predicted by the SDT. In South Korea and Taiwan, marriage and fertility rates have fallen to some of the lowest levels in the world, yet the prevalence of cohabitation and non-marital childbearing remains extremely limited (Lim, 2021; Cheng, 2023). Like Japan, both societies retain a strong cultural emphasis on parental duty and family lineage, and institutional frameworks continue to assume marriage as the normative foundation of family life.

In Singapore, the state's strong pro-family policies and emphasis on “responsible parenthood” have similarly reinforced a marriage-centered model, even as women's educational attainment and labor participation have risen sharply (Department of Statistics Singapore, 2024). Fertility decline has thus occurred within a policy environment that promotes individual aspiration while preserving traditional family norms—another example of the value–institution mismatch observed in Japan. In mainland China, the situation is somewhat distinct yet resonant. The expansion of women's educational and career opportunities has contributed to both later marriage and declining fertility. However, cohabitation and single parenthood remain socially marginal, constrained by legal, moral, and economic factors. Considering these factors, East Asian societies may share socioeconomic conditions associated with SDT, such as urbanization, gender equality in education, and post-materialist value changes; however, these have not yielded the same behavioral outcomes as in Western contexts due to cultural legacies, including long-standing family norms.

### 5.3 Theoretical and practical implications of the Child Care Commons (CCC)

Considering the generational and cultural patterns described above, this section discusses how emerging care arrangements might be interpreted in light of these broader dynamics. The results of Survey B also showed that many respondents stated that material resources, such as money, were important to them. These answers were primarily provided by individuals in their 20s and 30s, suggesting that young adults require both material and emotional support. Younger respondents in their 20s and 30s may hope that various family forms, including shared responsibility with non-family actors, will lessen the burden of child-rearing. Supporting this interpretation, younger age groups were less worried about sharing parental responsibility than older age groups in Survey A.

The concept of the CCC extends SDT debates by shifting attention from the forms of family to the processes of care. This may help explain why, even in the presence of value change, behavioral inertia persists, because care is embedded not only in individual preferences but also in relational norms. The CCC might play a critical role in reframing child-rearing not merely as a private duty of parents but as one of potential commons, illustrating how non-family actors might contribute to the reorganization of child-rearing.

The partial acceptance of CCC observed in the present study reflects both latent potential and risk. This tension suggests that for commons-based approaches to gain social legitimacy, institutional and policy support will be essential, for example, through formal recognition of shared care networks and gender-equitable redistribution of care work. Future research should examine how such commons-based approaches can be institutionally supported, particularly through policies.

### 5.4 Potential for use of ICTs for child-rearing

Although ICTs were not a central focus of the surveys, several responses suggested that they may become increasingly relevant for future child-rearing practices. The present results suggest a cautious attitude toward AI for family management policymaking, as indicated in Survey A (Q12) and comments in Surveys B and C. It is hard to imagine that, at current technological levels, people would place the same level of trust in an AI as they would in a family member, or that the AI would feel affection for the user. It is natural to resist having one's way of thinking restricted by such a system. Nevertheless, some younger respondents expressed expectations that ICTs might help ease certain burdens of child-rearing. The increase in administrative costs in a diversified society can be reduced through the appropriate use of AI and database technology.

Technological systems that allow selective sharing of information may help mitigate privacy concerns by enabling parents to disclose only what they choose at appropriate moments. AI technologies such as chatbots can, in principle, provide support without sharing personal information beyond the user, and ongoing discussions address how to ensure their safe use (Hasal et al., 2021). Beyond AI, modern database technologies such as blockchain have been explored for applications in healthcare, education, privacy protection, and the Internet of Things (Liu, 2016; Sharples and Domingue, 2016; Alketbi et al., 2018; Chen and Cho, 2021; Huynh-The et al., 2023), illustrating broader technological possibilities for improving privacy protection and reducing the costs of coordinating family-related activities. These technological developments suggest that ICTs may offer supplementary ways to support diversified child-rearing while addressing concerns related to privacy and responsibility.

### 5.5 Limitations and future directions

Several limitations of this study should be considered when interpreting the findings. First, this study examines normative attitudes rather than observed behavior; therefore, its findings should be interpreted as reflecting underlying value orientations that may precede or condition behavioral change rather than direct evidence of such change. Second, as fewer than 600 people participated in the present surveys, further research should be conducted to determine the generalizability of these findings. For example, the present study cannot answer the question of how differences in regions and/or income levels affect attitudes toward various family practices, given the current data from a limited number of individuals, which is too small to reveal differences among various and complex conditions. In Japan, non-standard family practices have been adopted in a limited number of regions. Regional differences in social networks may also exist. Considering these facts, clues that will lead to more accurate discussions may possibly be obtained by examining regional differences for a larger sample. However, because the survey was conducted across a wide range of regions and age groups, the results are unlikely to contain significant bias. Additionally, although this survey was conducted on PCs and smartphones, the high rate of Internet use in Japan makes it unlikely that the results were biased due to the use of online surveys. Longitudinal and comparative studies—especially across East Asian societies sharing similar family ideologies—would further clarify the interplay between value change and behavioral transformation.

## 6 Conclusion

This study examined how normative attitudes toward child-rearing in Japan relate to two central questions: (RQ1) the extent to which these attitudes correspond to or diverge from expectations associated with the Second Demographic Transition (SDT) theory, and (RQ2) the institutional and cultural constraints that may account for any observed divergences.

Regarding RQ1, our findings revealed a clear and systematic generational pattern across both structured evaluations and open-ended reflections. Younger adults expressed attitudes more consistent with SDT-related expectations, including greater openness to shared parental roles and to the involvement of non-family actors in child-rearing. In contrast, older adults adhered more strongly to traditional norms emphasizing exclusive parental responsibility and emotional–relational values such as affection, trust, and discipline. These results suggest a partial shift toward SDT-consistent orientations among younger cohorts, while traditional expectations remain salient among older adults.

Regarding RQ2, the analyses highlighted a set of persistent cultural expectations—particularly parental responsibility, privacy, and the boundary between family and non-family actors—that shape the limits of acceptance toward diversified child-rearing arrangements. Survey C, which introduced the Child Care Commons (CCC) as a hypothetical institutional innovation, made these constraints especially visible: although a majority of respondents evaluated the CCC concept positively, their free-text statements emphasized responsibility, privacy, and trust as central concerns. Moreover, willingness to personally participate in the CCC was more divided than general evaluations of the concept, indicating that these cultural expectations influence perceptions of practical feasibility as well as attitudes toward diversification.

Overall, the study demonstrates that generational differences and culturally embedded expectations jointly shape how Japanese people evaluate diversified child-rearing arrangements. By documenting both the areas of alignment with SDT-related expectations and the sources of divergence, these findings contribute concrete empirical evidence for refining theoretical accounts of family change in contemporary Japan and, more broadly, in East Asian societies. These discussions point to the relevance of examining how attitudes toward child-rearing, parental wellbeing, and role-based expectations intersect with institutional conditions. Further research building on this perspective may contribute to a better understanding of how parental wellbeing can be supported and how diverse forms of family practices can be sustained in contexts where behavioral change remains limited.

## Data Availability

The data supporting the conclusions of this article are not publicly available due to ethical and contractual restrictions, but may be made available from the corresponding author upon reasonable request.
