# Supplementary material for: General perception of the diversification of child-rearing environments in Japan
Source: Front Sociol. 2026 Mar 6;11:1674416. doi: 10.3389/fsoc.2026.1674416 (PMC13003535; doi:10.3389/fsoc.2026.1674416)
Supplement: Supplementary file 1 [file Data_Sheet_1.docx]

**Appendix**

**Supplementary Table S1.**
This table provides the detailed adjusted residuals and observed frequencies by age group for the chi-square analysis reported in Survey B (see Section 4.2 for detailed explanation).
